# Thymidine kinase as a biomarker of chemoresistance in epithelial ovarian cancer using the KELIM model

**DOI:** 10.3389/fphar.2026.1617484

**Published:** 2026-05-04

**Authors:** David Lukanović, Joško Osredkar, Erik Škof, Diana Cviič, Aleš Jerin, Kaja Lešnik, Miha Matjašič, Leon Meglič

**Affiliations:** 1 Department of Gynecology, Division of Gynecology and Obstetrics, Ljubljana University Medical Center, Ljubljana, Slovenia; 2 Department of Gynecology and Obstetrics, Faculty of Medicine, University of Ljubljana, Ljubljana, Slovenia; 3 Institute of Clinical Chemistry and Biochemistry, Ljubljana University Medical Center, Ljubljana, Slovenia; 4 Faculty of Pharmacy, University of Ljubljana, Ljubljana, Slovenia; 5 Department of Medical Oncology, Institute of Oncology Ljubljana, Ljubljana, Slovenia; 6 Medical Faculty, University of Ljubljana, Ljubljana, Slovenia; 7 Department of Education Studies, Faculty of Education, University of Ljubljana, Ljubljana, Slovenia; 8 Ginekologija Meglič d.o.o., Ljubljana, Slovenia

**Keywords:** biomarker, CA-125, chemoresistance, epithelial ovarian cancer, KELIM, ovarian cancer, thymidine kinase

## Abstract

**Background:**

Ovarian cancer (OC) remains the most lethal gynecological malignancy, with platinum sensitivity being a key determinant of treatment outcomes. The KELIM model, derived from CA-125 kinetics, is a promising biomarker for predicting chemosensitivity. Thymidine kinase 1 (TK1), a proliferation marker, has shown relevance in various cancers but its role in chemotherapy response for OC is unclear.

**Methods:**

In this retrospective study, we assessed the association between TK1 protein (TK1p) and enzymatic activity (TK1a) and chemosensitivity (KELIM), platinum-free interval (PFI), and chemotherapy response score (CRS) in 28 patients with epithelial OC treated with platinum-based chemotherapy. Biomarker dynamics were measured at multiple timepoints. KELIM was calculated using CA-125 kinetics; relationships with CRS and PFI were evaluated.

**Results:**

KELIM demonstrated robust predictive performance (correlating with favorable CRS [rho = 0.731, p = 0.011] and longer PFI [rho = 0.437, p = 0.007]). TK1p and TK1a showed no significant correlations with KELIM, PFI, or CRS. ROC analysis for preoperative TK1p yielded an AUC of 0.6941, indicating moderate discriminative potential. TK1a increased postoperatively but lacked predictive value for chemoresistance.

**Conclusion:**

Our findings reinforce the value of KELIM as a reliable predictor of platinum sensitivity in OC. TK1 dynamics reflect tumor proliferation but did not significantly predict chemotherapy response. Larger cohorts and further research are required to explore whether TK1 can complement established biomarkers.

## 1 Introduction

Ovarian cancer (OC) is the eighth most common malignancy affecting women worldwide and remains the most lethal among gynecological cancers. According to the Global Cancer Observatory, there were 313,959 new cases and 207,252 deaths globally in 2020 due to ovarian cancer (Sung et al., 2021). Alarmingly, more than two-thirds of patients are diagnosed at an advanced stage (FIGO stage III or IV), where the five-year survival rate drops to 27% and 13%, respectively. In contrast, early-stage diagnosis (FIGO stage I) significantly improves the five-year survival rate to over 90% (Menon et al., 2021; Han et al., 2018). These statistics highlight the urgent need for early detection, accurate prognostic markers, and reliable predictors of therapy response. Among the various histological types, high-grade serous carcinoma (HGSC) is the most common and aggressive subtype of epithelial OC. It is believed to arise from the epithelial cells of the fallopian tube fimbriae (National Comprehensive Cancer Network, 2024; Meinhold-Heerlein et al., 2016; Lisio et al., 2019). The standard primary treatment for HGSC includes primary debulking surgery (PDS), aimed at removing all visible tumor tissue, followed by adjuvant chemotherapy (ACT). For patients not suitable for upfront surgery or when optimal cytoreduction cannot be achieved, neoadjuvant chemotherapy (NACT) followed by interval debulking surgery (IDS) offers a viable alternative. Survival strongly depends on the extent of cytoreduction achieved, with complete resection being associated with the best outcomes. Despite advances in targeted therapies, platinum-based chemotherapy, most commonly carboplatin in combination with paclitaxel, remains the backbone of first-line treatment for advanced OC (Colombo et al., 2019). However, response to platinum therapy is variable: approximately 20%–40% of patients do not respond to initial treatment, and even among initial responders, up to 80% develop chemoresistant recurrence during follow-up (Lukanović et al., 2024). As platinum-resistant OC is nearly always fatal, platinum sensitivity remains the most important prognostic determinant in epithelial OC (Lukanović et al., 2024; Cornelison et al., 2017; Alberts et al., 2002).

To date, CA125 (MUC16) remains the most widely used biomarker for monitoring OC, especially for tracking treatment response and recurrence. However, its utility in early diagnosis is limited due to low sensitivity and specificity. CA125 can be elevated in several benign conditions, including endometriosis and pelvic inflammatory disease, reducing its diagnostic reliability [4,5]. To improve preoperative risk stratification, the Risk of Ovarian Malignancy Algorithm (ROMA) was introduced. It combines CA125 and human epididymis protein 4 (HE4) and is now routinely used in clinical practice (Li et al., 2012; Hada et al., 2020; Molina et al., 2011).

Despite these developments, no molecular predictive biomarkers for platinum resistance have been validated for routine use in clinical practice. This represents a critical gap in the management of OC, where early identification of patients unlikely to benefit from platinum therapy could allow for tailored treatment and potentially improved outcomes (Colombo et al., 2019). In this context, the KELIM model (CA-125 ELIMination rate constant K) has gained attention as a dynamic, quantitative marker of intrinsic tumor chemosensitivity. Studies have demonstrated that higher KELIM values are associated with improved platinum sensitivity and favorable outcomes, while low KELIM values indicate poor response to chemotherapy and higher risk of recurrence (National Comprehensive Cancer Network, 2024; Kim et al., 2023; You et al., 2021). KELIM is reproducible and has been validated in both frontline and recurrent disease settings, making it a promising tool for guiding treatment decisions (You et al., 2021). Another potential biomarker of interest is thymidine kinase 1 (TK1), a proliferation-associated enzyme involved in the DNA salvage pathway. TK1 is upregulated in proliferating cells, particularly during the S-phase of the cell cycle, and has been studied across various malignancies as a marker of tumor burden and cell proliferation. In OC, TK1 levels can be measured in serum either as enzyme activity (TK1a; U/L) or protein concentration (TK1p; μg/L). In our previous study by Cviič et al. (2023) we concluded that elevated TK1 levels correlate with tumor aggressiveness and have diagnostic and prognostic relevance, especially when used in combination with CA125 or HE4. However, its role in predicting chemotherapy response and platinum resistance remains poorly defined. Given the widespread need for accessible and dynamic predictors of treatment response, both KELIM and TK1 represent promising candidates for evaluation. While KELIM reflects tumor responsiveness to chemotherapy over time, TK1 may provide complementary information regarding tumor proliferative activity before and after treatment.

Therefore, the aim of this study was to investigate the relationship between pre- and post-treatment levels of TK1 (both activity and protein) and platinum sensitivity, as assessed by the KELIM model and Platinum free interval (PFI). By correlating TK1 with CA125, HE4, ROMA index, PFI and KELIM, we sought to evaluate the potential of TK1 as a predictive biomarker of platinum chemoresistance in epithelial OC, with the goal of contributing to more individualized treatment approaches.

## 2 Materials and methods

This retrospective observational study included patients with histologically confirmed epithelial OC who were treated at the Department of Gynaecology, University Medical Centre Ljubljana between 2020 and 2024. Although the primary focus was on patients with advanced-stage disease, a limited number of patients with early-stage but high-risk histological subtypes (e.g., high-grade serous or clear cell carcinoma or endometrioid carcinoma) were also included due to their higher likelihood of receiving systemic chemotherapy. All patients were treated with platinum-based chemotherapy, either in the adjuvant setting following PDS or after NACT followed by IDS. We excluded patients with a nonepithelial histologic type and patients with concomitant malignant neoplasms. In this retrospective observational study, we also excluded patients who received surgical treatment as the sole therapy for OC. The study design and methodology were based on the protocol previously described by Cviič et al. (2023) in their investigation of TK1 and OC. For our current study, the protocol was adapted to focus on patients with advanced epithelial OC undergoing chemotherapy, with specific modifications to the inclusion criteria and treatment setting to evaluate the prognostic value of these biomarkers—particularly TK1—in relation to platinum sensitivity assessed by the KELIM model. Clinical data, histopathological findings, biomarker results, treatment history, and follow-up information were collected from electronic medical records. The study was conducted in accordance with the ethical standards of the Declaration of Helsinki and was approved by the Slovenian National Medical Ethics Committee. All patients provided written informed consent for the use of their clinical data for research purposes.

Blood samples for biomarker analysis were collected at three distinct time points: before surgery, after surgery but prior to the initiation of chemotherapy, and after the completion of chemotherapy. Samples were processed using standard laboratory procedures, with serum aliquoted and stored at −80 °C until analysis. TK1 was measured in two forms, TK1p, expressed in μg/L, was assessed using the AroCell TK 210 ELISA, while TK1a, expressed in U/L, was measured using the LIAISON® TK assay. Both methods were performed in accordance with manufacturer protocols and established quality control standards. These biomarkers were analyzed at both baseline and post-treatment timepoints to evaluate their dynamic changes and potential prognostic significance.

Serum levels of CA125 and HE4 were measured using electrochemiluminescence immunoassays (ECLIA) on the Cobas e411 analyzer (Roche Diagnostics), according to the manufacturer’s instructions. The ROMA (Risk of Ovarian Malignancy Algorithm) index was calculated according to the standard algorithm using serum HE4 and CA125 concentrations (detailed in Supplementary Methods) (Cviič et al., 2023). We applied the CA-125 elimination rate constant K (KELIM) score to assess tumor response to chemotherapy. Using the online tool developed by You et al. (You et al., 2021; You et al., 2020). (https://www.biomarker-kinetics.org/CA-125-neo, accessed on 25 July 2024), we entered the date of each NACT cycle and the corresponding CA-125 levels recorded within the first 100 days of treatment. In line with the original study (You et al., 2021) we included at least the CA-125 values obtained prior to cycles 2, 3, and 4. The resulting output included the raw KELIM value and associated predictions for the probability of complete cytoreduction, platinum sensitivity, and overall survival. A higher KELIM value represents a faster decline in CA125 levels during treatment and thus reflects greater tumor chemosensitivity (You et al., 2021; You et al., 2020). In our analysis, the standardized KELIM values were dichotomized into two categories: unfavorable (KELIM < 1.0) and favorable (KELIM ≥ 1.0), based on previously validated thresholds. For tumor response to chemotherapy we looked also at PFI and Chemotherapy Response Score (CRS). PFI was defined as the time between the last dose of platinum-based chemotherapy and disease recurrence. Patients were categorized as platinum-sensitive (PFI > 12 months) or platinum-resistant (PFI < 12 months), in line with current clinical classification (National Comprehensive Cancer Network, 2024; Lukanović et al., 2024). CRS was used to assess histopathologic response to NACT. Based on the three-tiered system by Böhm et al. (2015), patients were classified as having a complete or near-complete response (CRS3), partial response (CRS2), or no/minimal response (CRS1) based on post-treatment histologic evaluation of omental and/or ovarian specimens (Böhm et al., 2015; Lawson et al., 2020; Cohen et al., 2019).

### 2.1 Statistical analysis

All statistical analyses were performed using R software (version 4.3.2) and the pROC package (version 1.17.0) for ROC curve analysis. Distributions of continuous variables were first inspected with Q–Q plots and the Shapiro–Wilk test (p < 0.05) which showed deviations from normal distributions for the principal biomarkers (KELIM std, CRS, CA-125, HE-4, TK1 activity and TK1 protein). Furthermore Platinum-free interval (PFI) was dichotomised *a-priori* as platinum-resistant (<12 months, code 0) versus platinum-sensitive (>12 months, code 1) and Standardised KELIM was categorised as unfavourable (<1.0) or favourable (≥1.0) in keeping with the original validation study. Missing values were excluded and analyses that involved CRS were restricted to the eleven individuals for whom a score was available.

In the next step, descriptive statistics were calculated and presented in the form of frequencies and percentages and, for continuous variables, also as mean and standard deviation.

Changes in biomarker values within the patient group at the three time points (preoperative, postoperative/pre‐chemotherapy and post‐chemotherapy) were analyzed using the Wilcoxon signed‐rank test.

In order to assess correlations between two continuous variables, they were quantified with Spearman’s rank correlation coefficient (ρ); when tied ranks were prevalent, Kendall’s τ was preferred. Moreover when assessing correlation between PFI and continuous variable, the association was summarized with the rank-biserial correlation (r_rb_). Finally in order to examine the discriminative ability of pre-operative TK1 to separate favorable from unfavorable KELIM status, non-parametric receiver-operating-characteristic curves were constructed and the area under the curve (AUC) was estimated with DeLong’s method.

All statistical tests were two‐sided, and p < 0.05 was considered statistically significant.

## 3 Results

A total of 28 patients with epithelial OC were included in the analysis. All patients received platinum-based chemotherapy, either following PDS or as part of NACT followed by IDS. The summarized patient data and descriptive statistics for all measured biomarkers are presented in Table 1.

**TABLE 1:** Patient characteristic.

​	N	%	Mean	Std. Deviation
Age	28	100	65.29	11.97
Menopausal status	​	​	​	​
• Premenopausal	6	21.4	​	​
• Postmenopausal	22	78.6	​	​
Histology	​	​	​	​
• High grade serous	20	71.43	​	​
• Low grade serous	3	10.71	​	​
• Clear cell	1	3.57	​	​
• Endometrioid	4	14.29	​	​
FIGO stage	​	​	​	​
• I, II	6	21.44	​	​
• III.A	1	3.57	​	​
• III.B	3	10.71	​	​
• III.C	15	53.57	​	​
• IV.A	2	7.14	​	​
• IV.B	1	3.57	​	​
TK1a	​	​	​	​
• Before primary surgery	28	100	11.61	7.45
• After primary surgery	22	79	27.46	28.80
• After chemotherapy	27	96.43	16.21	15.59
TK1p	​	​	​	​
• Before primary surgery	28	100	0.87	0.60
• After primary surgery	22	79	0.92	0.74
• After chemotherapy	27	96.43	0.64	0.46
CA-125	​	​	​	​
• Before primary surgery	28	100	825.70	1232.97
• After chemotherapy	26	92.86	31.22	57.05
HE 4	​	​	​	​
• Before primary surgery	28	100	587.80	566.43
• After chemotherapy	26	92.86	140.40	139.28
Roma index (before primary surgery)	28	100	74.22	28.67
• Premenopausal women	​	​	​	​
o High risk of malignancy: ROMA ≥ 13.1%	6	100	​	​
o Low risk of malignancy: ROMA < 13.1%	0	0	​	​
• Postmenopausal women	​	​	​	​
o High risk of malignancy: ROMA ≥ 27.7%	19	86.37	​	​
o Low risk of malignancy: ROMA < 27.7%	3	13.63	​	​
Roma index (after chemotherapy)	26	92.86	26.81	26.04
• Premenopausal women	​	​	​	​
o High risk of malignancy: ROMA ≥ 13.1%	1	16.67	​	​
o Low risk of malignancy: ROMA < 13.1%	4	66.67	​	​
• Postmenopausal women	​	​	​	​
o High risk of malignancy: ROMA ≥ 27.7%	8	36.36	​	​
o Low risk of malignancy: ROMA < 27.7%	13	59.09	​	​
KELIM score	​	​	​	​
• <1	18	64.29	​	​
• ≥1	10	35.71	​	​
PFI	​	​	​	​
• Platinum-resistant disease; PFI < 12 months	7	25.00	​	​
• Platinum-sensitive disease; PFI > 12 months	21	75.00	​	​

FIGO, international federation of gynaecology and obstetrics; TK1a, TK1 activity; TK1p, TK1 protein; KELIM, CA125 elimination rate constant K; PFI, platinum free interval.

### 3.1 Correlation between biomarkers and tumor responses to chemotherapy

**FIGURE 1:**
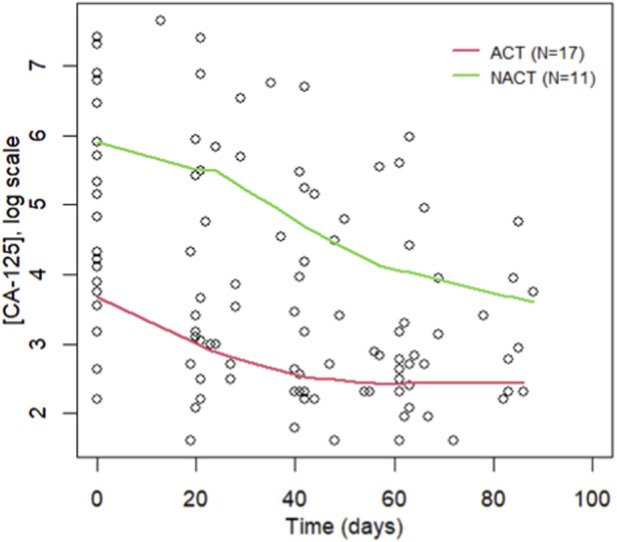
CA-125 Kinetics Over the First 100 Days of Chemotherapy Used for KELIM Calculation (ACT, adjuvant chemotherapy; NACT, neoadjuvant chemotherapy).

The mean KELIM value was 0.96 (range: 0.49–1.97), with a median of 0.98. As shown in Figure 1, the ACT group (red) demonstrates a steeper and more consistent decline in CA-125 levels during the first 100 days of chemotherapy, indicating a faster CA-125 elimination rate and suggesting higher intrinsic tumor chemosensitivity. In contrast, the NACT group (green) exhibits a slower and more variable decline, consistent with reduced tumor marker clearance and potentially lower chemosensitivity.

Statistically significant correlations were observed between key clinical, dynamic, and preoperative biomarker parameters. Spearman’s rank correlation showed strong positive association between CRS and standardized KELIM values (ρ = 0.731, *p* = 0.011, *n* = 11). Using the non-parametric rank-biserial correlation, KELIM std showed strong positive association with platinum-free interval (r_rb_ = 0.70, p = 0.007; n = 28). However, no significant association was found between CRS and platinum-free interval (r_rb_ = −0.30, p = 0.38, n = 11) Regarding preoperative serum markers, CA-125 and HE-4 showed significant negative correlation with PFI (CA-125 r_rb_ = −0.73, p = 0.005; HE-4 r_rb_ = −0.54, p = 0.035 A strong positive correlation was also observed between CA-125 and the ROMA index (τ = 0.647, *p* < 0.001), and between HE4 and the ROMA index (τ = 0.831, *p* < 0.001), with a moderate positive correlation noted between CA-125 and HE4 (τ = 0.516, *p* < 0.001). Although weak negative correlations were found between KELIM and both the ROMA index (τ = −0.212, *p* = 0.114) and HE4 (τ = −0.229, *p* = 0.091), these did not reach statistical significance. Additional positive associations were noted between CA-125 and HE4 (τ = 0.320, *p* = 0.039), CA-125 and ROMA index (τ = 0.495, *p* = 0.001), and HE4 and ROMA index (τ = 0.800, *p* < 0.001).

In the post-treatment phase, similar patterns were observed. Post-treatment CA-125 remained significantly correlated with both the ROMA index (τ = 0.495, *p* = 0.001) and HE4 (τ = 0.320, *p* = 0.039). HE4 and ROMA index also showed a strong correlation (τ = 0.800, *p* < 0.001). Notably, KELIM retained a significant negative correlation with post-treatment CA-125 (τ = −0.377, *p* = 0.014), supporting its role as an early dynamic marker of treatment response. However, its correlations with post-treatment HE4 (τ = −0.197, *p* = 0.203) and ROMA (τ = −0.252, *p* = 0.102) were not statistically significant.

### 3.2 Thymidine kinase

TK1 was measured at three time points: before primary surgery, after surgery/before chemotherapy, and after chemotherapy. The preoperative TK1 activity (TK1a) had a mean of 11.62 U/L and TK1 protein (TK1p) 0.87 μg/L. Postoperatively, TK1a increased (mean 27.46 U/L), while TK1p was relatively stable (mean 0.92 μg/L). Following chemotherapy, a decline in TK1 was observed: TK1a averaged 16.21 U/L and TK1p 0.64 μg/L.

A Wilcoxon signed-rank test was used to assess changes in TK1 levels over the course of treatment. While there was a trend toward decreased TK1p after chemotherapy in 16 patients and increased values in 10, the difference was not statistically significant (Z = −1.207, p = 0.228). Similarly, for TK1a, 11 patients had lower post-chemotherapy values compared to preoperative levels, and 16 had increased values, but again the difference was not statistically significant (Z = −1.201, p = 0.230).

The longitudinal evaluation of TK1 biomarkers revealed distinct trends between platinum-sensitive and platinum-resistant patient groups across the treatment course (Table 2). Groups were divided according to KELIM score (≥1 or <1). TK1a (U/L) increased from pre-treatment to post-treatment in both groups, with a more pronounced rise in the platinum-resistant group (13.30 → 21.80 U/L) compared to the platinum-sensitive group (10.39 → 16.30 U/L). Although TK1a declined at follow-up, it remained above baseline in both groups, indicating sustained cellular proliferation. The relative decrease from pre-treatment to follow-up was modest and comparable between groups (−4.11% vs. −3.8%).

**TABLE 2:** Median values for biomarkers in platinum-sensitive and platinum-resistant groups.

Biomarker	Group	Pre-surgery	Post- surgery	Post- CT	Δ (pre → post surgery)	Δ (pre → post CT)
TK1a (U/L)	Platinum-sensitive	10.39	16.30	14.50	−5.91	−4.11
​	Platinum-resistant	13.30	21.80	17.10	−8.5	−3.8
TK1p (μg/L)	Platinum-sensitive	0.60	0.68	0.48	−0.08	0.12
​	Platinum-resistant	0.73	0.65	0.38	0.08	0.35
CA125 (U/mL)	Platinum-sensitive	277.60	129.80	10.70	147.8	266.9
​	Platinum-resistant	1831.0	747.30	29.80	1083.7	1801.2
HE4 (pmol/L)	Platinum-sensitive	375.40	138.60	72.80	236.8	302.6
​	Platinum-resistant	955.00	559.80	207.80	395.2	747.2
ROMA index (%)	Platinum-sensitive	86.00	60.10	13.20	25.9	72.8
​	Platinum-resistant	96.70	96.70	48.60	0	48.1

Median values are used for biomarker levels; Δ calculations highlight changes over time for trend analysis; TK1a, TK1 activity; TK1p, TK1 protein; CT, chemotherapy.

In contrast, TK1p (μg/L) exhibited divergent temporal patterns. Among platinum-sensitive patients, TK1p rose post-surgery and declined after chemotherapy (0.60 → 0.68 → 0.48 μg/L). In platinum-resistant patients, however, TK1p showed a continuous decrease across all timepoints (0.73 → 0.65 → 0.38 μg/L). Notably, the overall change from baseline to follow-up was more substantial in the resistant group (Δ = 0.35 μg/L) than in the sensitive group (Δ = 0.12 μg/L), suggesting differential regulation of TK1p expression in response to chemotherapy.

**FIGURE 2:**
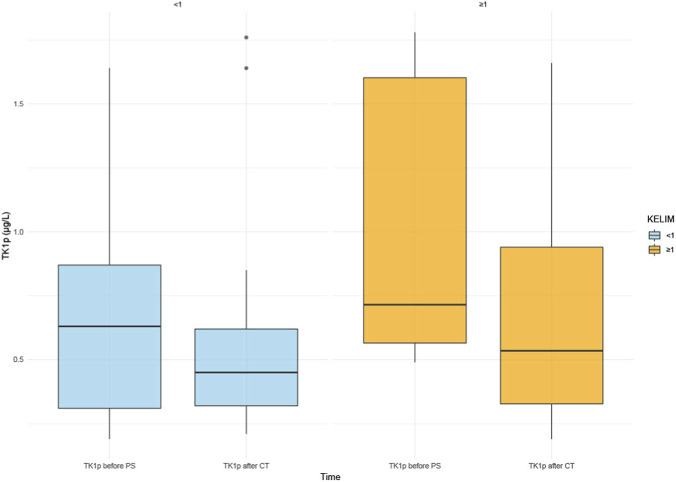
Comparison of TK1p Before primary surgery and After Chemotherapy in Platinum-Sensitive (n = 18) and Platinum-Resistant (n = 10) Ovarian Cancer Patients (TK1a, TK1 activity; TK1p, TK1 protein; PS, primary surgery; CT, chemotherapy; KELIM, CA125 elimination constant K).

Based on the descriptive statistics (Table 2), the unfavorable group shows higher mean and median values for TK1p before treatment (mean 1.01 vs. 0.686, median 0.715 vs. 0.63) and also slightly higher values after treatment (mean 0.71 vs. 0.595, median 0.535 vs. 0.45) - Figure 2. However, the Wilcoxon tests showed p-values of approximately 0.1026 for the pre-treatment values and 0.6151 for the post-treatment values, which means these differences were not statistically significant at the 0.05 threshold.

### 3.3 Correlation of TK1 with KELIM

**FIGURE 3:**
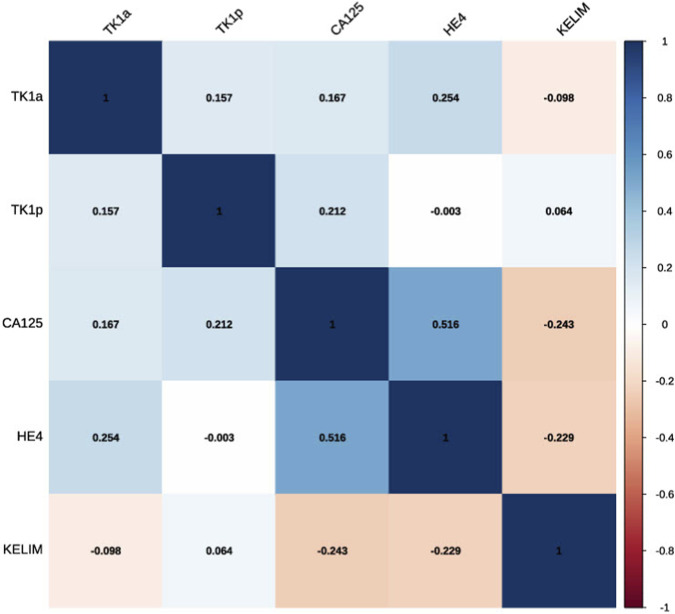
Kendalls T Correlation Matrix (TK1a, TK1 activity; TK1p, TK1 protein; KELIM, CA125 elimination constant K).

Correlation analyses between TK1 biomarkers and treatment response indicators showed no statistically significant associations (Figure 3). Preoperative TK1a (U/L) demonstrated a weak negative correlation with KELIM (τ = −0.098, *p* = 0.465) and a modest negative correlation with platinum-free interval (PFI) (r_rb_ = −0.442, *p* = 0.090), neither of which reached statistical significance. Similarly, TK1p (μg/L) measured preoperatively showed no significant correlation with KELIM (τ = 0.064, *p* = 0.635) or PFI (r_rb_ = −0.21, *p* = 0.426).

Postoperative and post-chemotherapy TK1 values also lacked significant correlations with KELIM. TK1a measured after surgery showed only a very weak positive association with KELIM (τ = 0.143, *p* = 0.352), while TK1p postoperatively had a slightly stronger but still nonsignificant correlation (τ = 0.091, *p* = 0.553). TK1 activity and protein levels measured after chemotherapy exhibited weak correlations with KELIM (τ = 0.066 and τ = 0.222, respectively), with the latter nearing but not reaching statistical significance (*p* = 0.108). The only statistically significant correlation observed was between postoperative TK1 activity and protein levels (τ = 0.570, *p* < 0.001), indicating a consistent intra-biomarker correlation post-surgery. Nevertheless, no significant correlations were found between TK1 (both TK1a and TK1p) and KELIM at any treatment stage.

### 3.4 ROC analysis

**FIGURE 4:**
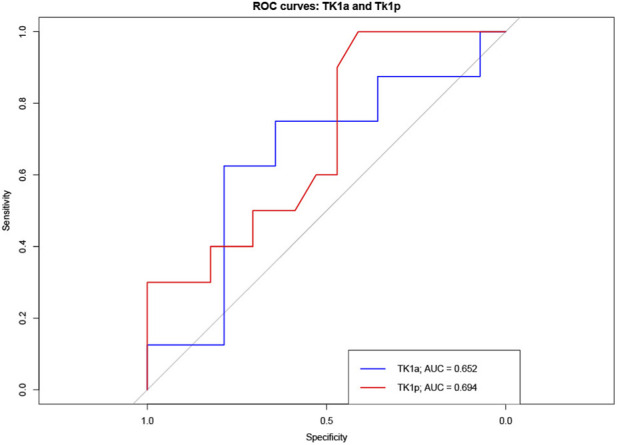
ROC curve analysis for TK1p (red) and TK1a (blue) in predicting KELIM-defined chemosensitivity (n = 28). Area under the curve (AUC) values are indicated.

ROC analysis (Figure 4) was performed to assess the ability of TK1a and TK1p to predict tumor chemosensitivity, defined by KELIM status (KELIM < 1 vs. ≥ 1). TK1p measured preoperatively showed moderate discriminative power, with an AUC of 0.6941, correctly classifying KELIM status in approximately 69.4% of cases, indicating moderate discriminative potential. In comparison, TK1a measured prior to surgery demonstrated a lower AUC of 0.60, indicating only modest predictive performance.

Biomarker time-course dynamics across treatment are shown in the Supplementary Figure. Median values of TK1a activity (Panel A, U/L), TK1p protein (Panel B, μg/L), and CA-125 (Panel C, U/mL) are presented at three treatment timepoints: pre-operative baseline (Pre-Surgery), post-operative pre-chemotherapy (Post-Surgery), and post-chemotherapy (Post-CT). Red lines represent platinum-sensitive patients (PS, KELIM ≥ 1.0, n = 21), and blue lines represent platinum-resistant patients (PR, KELIM < 1.0, n = 7). Sample sizes at each timepoint are indicated in parentheses. TK1a and TK1p demonstrated distinct temporal patterns between groups, while CA-125 kinetics clearly delineated platinum sensitivity status.

## 4 Discussion

Despite advancements in molecular diagnostics and targeted therapies, predicting response to platinum-based chemotherapy remains a central challenge in the management of epithelial OC. As nearly one-third of patients exhibit intrinsic resistance and most eventually relapse, reliable tools to assess tumor chemosensitivity are critical for guiding treatment strategies. To address this gap, our study explored the utility of a dynamic mathematical parameter—KELIM—as well as the proliferation marker TK1 in evaluating treatment response. Our findings offer new insights into how these approaches reflect distinct dimensions of tumor biology, with implications for individualizing care and optimizing outcomes. The study’s findings support the KELIM model’s predictive ability to evaluate platinum sensitivity and tumor chemosensitivity in epithelial OC. Our results reinforce KELIM’s role as a therapeutically meaningful biomarker for guiding chemotherapy strategies by confirming that higher KELIM values are associated with improved treatment responses—consistent with previous studies. Furthermore, although tumor burden may influence early chemosensitivity, KELIM appears to remain an independent predictor of platinum response, as reflected by the modest negative correlations observed between KELIM and preoperative tumor markers (CA125, HE4, ROMA index).

Using data from the ICON8, CHIVA, and AGO OVAR trials, the KELIM model demonstrated excellent predictive accuracy in both NACT and ACT. Parameters derived from ICON8 and CHIVA were used for the NACT cohort, while AGO OVAR data informed the ACT group (You et al., 2020). Observed CA-125 values closely aligned with individual model predictions, and normally distributed residuals confirmed statistical robustness. Visual predictive checks demonstrated that CA-125 kinetics consistently fell within predicted confidence intervals, validating the model’s ability to reflect tumor chemosensitivity over time.

These results demonstrate the potential of KELIM as a useful biomarker for evaluating the response of tumors to treatment. KELIM enables improved therapeutic approach classification by reflecting early tumor shrinkage kinetics and offering an objective way to identify patients with high chemosensitivity.

The predictive importance of KELIM is further supported by comparison with earlier research. Higher KELIM levels have been linked to better overall survival (OS) and progression-free survival (PFS) in ovarian cancer, according to several studies. A research by You et al. (2021), for instance, found that KELIM was an independent predictor of platinum sensitivity, supporting our findings that improved postoperative outcomes are associated with higher KELIM levels.

While weak negative correlations were found between KELIM and ROMA index, CA125, and HE4 in the preoperative setting, they did not reach statistical significance. This may reflect biological heterogeneity in tumor marker kinetics or limitations due to sample size. However, a statistically significant moderately negative correlation was observed between KELIM and postoperative CA125 (τ = −0.377, p = 0.014), suggesting that KELIM more accurately represents tumor chemosensitivity following cytoreductive surgery. The observed interrelationships among ROMA index, HE4, and CA125 also reaffirm the interdependence of these markers in OC prognosis.

Although CA125 dynamics have previously been proposed as a surrogate marker for treatment response, its predictive capacity is inferior to that of KELIM. A decrease in CA125 does not always indicate tumor chemosensitivity, which has led some studies to suggest that combining CA125 kinetics with KELIM may improve response prediction (You et al., 2020; You et al., 2013; Wilbaux et al., 2014; You et al., 2023; Bouvarel et al., 2024; Colomban et al., 2019).

Analysis of TK1 dynamics revealed distinct patterns in TK1 protein (TK1p) and TK1 activity (TK1a) across the treatment timeline: (Sung et al., 2021): TK1a levels were relatively low prior to surgery; (Menon et al., 2021); TK1a increased markedly after surgery, likely reflecting either release from dying tumor cells or reactive proliferation; (Han et al., 2018); both TK1a and TK1p declined post-chemotherapy, consistent with reduced tumor cell proliferation following treatment.

Although the majority of patients showed a trend toward reduced TK1p levels after chemotherapy, the change was not statistically significant. This may be due to inter-patient variability in chemosensitivity, the presence of residual disease, or differences in TK1 response kinetics.

Our findings align with earlier studies reporting increased TK1 activity after surgery, potentially indicating ongoing tumor activity or tissue remodeling (N et al., 2010; Pan et al., 2021). However, unlike some prior reports, we observed variable TK1 responses across patients, with less consistent post-chemotherapy declines.

Notably, no significant correlations were found between TK1 (both TK1a and TK1p) and KELIM at any treatment stage. This suggests that TK1 may not be a reliable surrogate marker for early chemosensitivity. Nonetheless, from a biological standpoint, higher TK1 activity may reflect increased DNA synthesis and cell proliferation. TK1 is a key enzyme in the salvage pathway of pyrimidine nucleotide synthesis, predominantly expressed during the S-phase of the cell cycle. Under normal conditions, TK1 activity is tightly regulated. In cancer, however, dysregulated cell cycle control—particularly disruption of the G1/S checkpoint—can lead to sustained or elevated TK1 expression. Thus, increased TK1 activity in serum or tumor tissue may indicate a high proliferative index, impaired cell cycle regulation, and genomic instability—all features commonly associated with aggressive tumor behavior and therapeutic resistance. However, the magnitudes of the observed correlations argue against a clinically relevant link between TK1 dynamics and intrinsic platinum sensitivity.

In our recent study by Cviič et al. (2023), we demonstrated that both TK1 protein and activity levels are elevated in ovarian cancer and correlate with tumor burden and proliferative capacity. However, their utility in predicting chemotherapy response appears limited when compared to more established dynamic markers such as CA125.

Interestingly, TK1a and TK1p showed a significant correlation in the preoperative phase (τ = 0.570, p < 0.001), suggesting that these two measures are tightly linked under baseline conditions. Yet, the lack of correlation between TK1 and other tumor markers (CA125, HE4, ROMA index) suggests that TK1 reflects a distinct biological pathway—namely, cell proliferation—rather than overall tumor burden or platinum sensitivity.

Although TK1 has been identified as a promising proliferation marker in several malignancies, its role in OC remains uncertain. Some studies have reported limited predictive value in this setting, while others have shown correlations with Ki-67, another marker of cell proliferation (Wang et al., 2017; Bitter et al., 2020; Rödel et al., 2020).

The magnitudes of the observed correlations (<|0.30|) argue against a clinically relevant link between TK1 dynamics and intrinsic platinum sensitivity as captured by KELIM in this dataset. However, ROC analysis further supported the diagnostic value of TK1 in identifying chemosensitive tumors. TK1 measured preoperatively demonstrated moderate discriminative ability for predicting tumor chemosensitivity based on KELIM status, with an AUC of 0.6941. This implies that TK1 correctly classified KELIM status in approximately 69.4% of cases. Although this level of discrimination is moderate rather than strong, it indicates some predictive value. In contrast, TK activity measured before chemotherapy) showed weaker performance, with an AUC of 0.60, suggesting only modest predictive utility.

The superior ROC curve of preoperative TK1, which ascended steeply and approached the top-left of the plot space, reflects its better sensitivity and specificity profile compared to TK activity. These findings support the hypothesis that TK1 protein levels might provide moderate value in differentiating patients by chemosensitivity. However, neither marker achieved high diagnostic accuracy, underscoring the need for multimarker approaches - for example, combining TK1 with other biomarkers such as KELIM or CA125 for optimal predictive stratification.

Clinical implications; (Sung et al., 2021); The use of KELIM into clinical decision-making is supported by its noteworthy predictive value. Alternative or more intensive therapeutic approaches, such as targeted medicines or maintenance medications, may be necessary for patients with low KELIM scores (Menon et al., 2021). Postoperative CA125 levels may be used as an additional marker to evaluate tumor chemosensitivity, as indicated by the moderately negative correlation found between them and KELIM (Han et al., 2018). Although changes in TK1a indicate changes in the dynamics of tumor proliferation, its lack of association with KELIM and conventional tumor markers calls into question its use as a prognostic biomarker for OC. This study’s primary limitation is the modest sample size, further reduced for CRS analysis (n = 11). As a result, the statistical power is limited, and generalizability is constrained. To fully understand its usefulness, more research with bigger cohorts is required (National Comprehensive Cancer Network, 2024). The post-treatment changes in TK1a and TK1p dynamics show that more research is necessary to ascertain whether TK1 fluctuations could be used to identify patients who are more likely to experience a recurrence.

A strength of this study is the accurate modeling of CA125 kinetics, as the results highlight the stronger predictive value of KELIM following cytoreductive surgery, which may improve patient stratification for subsequent therapy. In addition, the study included a comprehensive biomarker analysis incorporating CA125, HE4, the ROMA index, and TK1, allowing for a broader evaluation of tumor biology and treatment response. The analysis of TK1 dynamics further contributes novel observations regarding its behavior during treatment, despite the lack of a significant correlation with KELIM. However, several limitations should be acknowledged. The relatively small sample size, including only 28 patients and a subgroup of 11 patients with available cytoreductive surgery data, limits statistical power and reduces the ability to detect more subtle associations, thereby affecting generalizability. The single-center study design also restricts external validity, although it ensured uniform treatment protocols and consistent sample handling; this is particularly relevant in Slovenia, where advanced ovarian cancer treatment is concentrated in only three centers serving a population of approximately two million. Furthermore, although KELIM reflects early chemosensitivity, longer follow-up is required to determine its impact on progression-free and overall survival. Variability in TK1 response following treatment suggests the presence of potential unmeasured confounders, such as differences in residual disease burden. Interpretation of the predictive performance of KELIM is additionally limited by the absence of direct comparison with alternative chemosensitivity models, and biomarker dynamics may also have been influenced by variability in prior treatments, including differences in chemotherapy regimens that were not fully explored in this study.

## 5 Conclusion

In evaluating tumor chemosensitivity in OC, particularly following cytoreductive surgery, this study validates the prognostic value of the KELIM model. Its potential for therapy stratification is further supported by the observed associations between KELIM and CA125 kinetics. While TK1a dynamics suggest a role in tumor proliferation, their lack of correlation with chemotherapy response limits their predictive utility, indicating that TK1 may reflect biological processes distinct from chemosensitivity. Although TK1 activity followed the expected pattern of postoperative increase and post-chemotherapy decline, its prognostic significance remains uncertain.

With all factors considered, our results demonstrate the therapeutic value of KELIM as a reliable gauge of early chemosensitivity and highlight the need for future studies with larger cohorts and longer follow-up to clarify the clinical role of TK1. Although TK1 and KELIM capture distinct aspects of tumor biology, their potential complementary role in predicting platinum sensitivity should be explored in larger, prospective studies.

## Data Availability

The datasets generated and analyzed during the current study are available from the corresponding author upon reasonable request.
