# Supplementary material for: Thymidine kinase as a biomarker of chemoresistance in epithelial ovarian cancer using the KELIM model
Source: Front Pharmacol. 2026 May 4;17:1617484. doi: 10.3389/fphar.2026.1617484 (PMC13180823; doi:10.3389/fphar.2026.1617484)
Supplement: Supplementary file 1 [file Supplementaryfile1.docx]

**Supplementary Methods: Calculation of the Risk of Ovarian Malignancy Algorithm (ROMA)**

The Risk of Ovarian Malignancy Algorithm (ROMA) was calculated based on serum concentrations of **human epididymis protein 4 (HE4)** and **cancer antigen 125 (CA125)** according to the formulas originally described by Moore et al. (2009). Separate predictive index (PI) equations were used for **premenopausal** and **postmenopausal** women:

$$\text{Premenopausal}\text{: }PI=-12.0+2.38\times\ln(\text{HE4})+0.0626\times\ln(\text{CA125})$$

$$\text{Postmenopausal: }PI=-8.09+1.04\times\ln(\text{HE4})+0.732\times\ln(\text{CA125})$$

The **ROMA value** (expressed as a percentage risk) was then calculated as:

$$ROMA=\frac{e^{PI}}{1+e^{PI}}\times100$$

Cutoff values were applied as follows:

- **Premenopausal women:** ROMA ≥ 13.1% indicates high risk of malignancy.
- **Postmenopausal women:** ROMA ≥ 27.7% indicates high risk of malignancy.

Serum HE4 and CA125 levels were measured using commercially available immunoassays according to the manufacturer’s instructions. All calculations were performed using the natural logarithm (ln) of the measured concentrations.

**Reference:**

Moore RG, McMeekin DS, Brown AK, DiSilvestro P, Miller MC, Allard WJ, Gajewski W, Kurman R, Bast RC Jr, Skates SJ. A novel multiple marker bioassay utilizing HE4 and CA125 for the prediction of ovarian cancer in patients with a pelvic mass. Gynecol Oncol. 2009 Jan;112(1):40-6. doi: 10.1016/j.ygyno.2008.08.031.

Jeong TD, Cho EJ, Ko DH, Lee W, Chun S, Kwon HJ, Hong KS, Kim YM, Min WK. A new strategy for calculating the risk of ovarian malignancy algorithm (ROMA). Clin Chem Lab Med. 2017 Jul 26;55(8):1209-1214. doi: 10.1515/cclm-2016-0582.

**Supplementary Figure: Biomarker Time Course Dynamics Across Treatment**

**
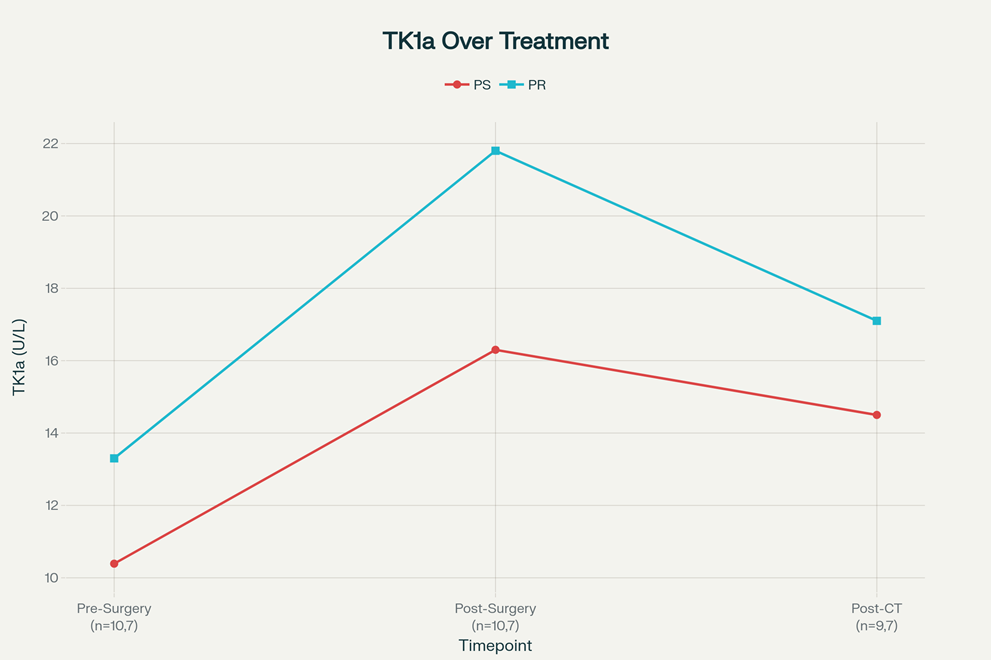
**

Supplementary Figure: Biomarker Time-Course Dynamics Across Treatment. Three-panel figure showing median values of (A) TK1a activity (U/L), (B) TK1p protein (µg/L), and (C) CA-125 (U/mL) in platinum-sensitive (PS, red, KELIM ≥1.0) and platinum-resistant (PR, blue, KELIM <1.0) patients at three treatment timepoints. Sample sizes (n) are indicated for each group and timepoint. PS group: n=21; PR group: n=7
